# You are simply not funny: Development and validation of a scale to measure failed humor in leadership

**DOI:** 10.3389/fpsyg.2022.929988

**Published:** 2022-07-22

**Authors:** Alexander Pundt, Juana Kutzner, Katarina Haberland, Mona Algner, Timo Lorenz

**Affiliations:** Department of Psychology, Medical School Berlin (MSB), Berlin, Germany

**Keywords:** humor in leadership, failed humor, scale development, meta heuristic algorithm, scale validation

## Abstract

Research has recently established the notion that humor in leadership contributes to the development of a positive professional relationship between leaders and followers. This relationship has been supposed to be the core mechanism *via* which humor in leadership unfolds its effects on work attitudes and behaviors. However, research has neglected the option that humor used by leaders might fail to amuse their followers. In this study, we investigate the role of failed humor for the relationship between leader and follower. More concretely, we develop a new scale for measuring failed humor in leadership and demonstrate its factorial and criterion-related validity. Using an automated item selection algorithm, we optimized the newly developed scale and derived a well-fitting six-item scale out of a pool of 12 items. In a study based on a sample of 385 employees, we were able to show that our newly developed scale is factorially valid. Moreover, we showed a negative correlation between failed humor and leader-member exchange. Furthermore, we showed incremental validity of failed humor in that failed humor predicted variance in leader-member exchange beyond well-established humor constructs such as affiliative and aggressive humor. Our study contributes to the development of the field of humor in leadership and opens up new options for further inquiry. Moreover, our study demonstrates the use of automated item selection algorithms in the applied field.

## Introduction

Recent research has demonstrated the usefulness and positive consequences of humor in leadership (Kong et al., 2019). Although some studies show the necessity to distinguish between several forms of humor such as affiliative and aggressive humor (Martin et al., 2003; Pundt and Herrmann, 2015; Pundt and Venz, 2017) or, more recently, the so-called comic style markers (e.g., Ruch et al., 2018), the overarching assumption of research on humor in leadership seems to be that the humor used by a leader actually unfolds the intended effects. This, however, clearly contradicts common knowledge after which humor—in particular humor used by a leader—might very well fail to unfold its intended effects. Besides some linguistic, pragmatic research on failed humor (Bell, 2015; Brock, 2016; Dynel et al., 2016), evidence on failed humor in the context of organizations and leadership is rather sparse. The authors are aware of just one study: In their study, Williams and Emich (2014) take the perspective of leaders using humor but failing to reach the intended effects—in this case interpersonal affect regulation. Bell (2015, p. 1) points out that failed humor can have “serious social consequences” beyond the fact that the receiver of humor is simply not amused. For example, after occasions of failed humor, the receiver’s first impression of the sender of failed humor might be rather negative. Consequently, this negative relationship might be detrimental for the development of a positive personal relationship between sender and receiver.

In the context of leadership, this is particularly worrisome as research has demonstrated that the main effect of humor in leadership (Kong et al., 2019) and leadership behavior in general (Gottfredson and Aguinis, 2017) is mediated *via* leader-member exchange, which is an indicator of the quality of the leader-follower relationship (e.g., Graen and Uhl-Bien, 1995). In other words: Failed humor in leadership might be detrimental for establishing a positive professional relationship between leader and follower and might, therefore, have negative consequences for desired leadership outcomes such as performance or wellbeing (e.g., Dulebohn et al., 2012; Montano et al., 2017). It is, therefore, surprising that failed humor in leadership is rarely investigated.

In this study, we attempt to initially investigate failed humor in leadership. We define and further elaborate the concept of failed humor in leadership and, in a next step, develop and examine a measure and demonstrate its relation with leader-member exchange. Furthermore, we show the incremental validity of failed humor in comparison to affiliative and aggressive humor in leadership as well-established constructs in humor research (Martin et al., 2003; Pundt and Herrmann, 2015).

This research might contribute to the literature in at least three ways: First, by introducing the concept of failed humor (Bell, 2015) to research on humor in leadership and demonstrating its incremental validity beyond other humor styles, we widen the perspective on humor in leadership and help acknowledge that humor might fail to unfold its effects. Doing so, we underline the empirical validity of the common notion that humor might fail and suggest a measure for assessing failed humor in leadership. In a more general way, we contribute to knowledge on communication between leaders and followers. In line with Bell (2015), failed humor can be seen as a form of misunderstanding between leaders and followers. Our research demonstrates that such misunderstanding might be detrimental for the relationship between the sender (i.e., the leader) and the receiver (i.e., the follower) of failed humor. With respect to leadership research, we aim to show that well-intended leader behavior might fail to have its intended effects and unfold negative or at least unintended side effects (see also Pundt, 2014 for a similar problem).

Looking back at previous work (e.g., Craik et al., 1996), one might wonder why we attempt to develop a new measure for assessing failed humor when the Humor Behavior Q-sort Deck by Craik et al. (1996) contains a subscale called inept humor. At first glance, there seem to be conceptual similarities between our idea of failed humor and inept humor in that inept humor claims to assess the inability to tell jokes effectively (Craik et al., 1996). However, upon review of the respective items, it becomes clear that the inept humor scale is more focused on the receiver side of humor rather than on the sender side. Therefore, the idea of failed humor still waits for being empirically addressed adequately.

## The (successful) process of humor in leadership

In line with other definitions of humor (e.g., Cooper, 2005; Romero and Cruthirds, 2006; Martin, 2007; Robert and Wilbanks, 2012), we define humor in leadership as a discretionary social behavior, the core of which is a playful (non-)verbal activity initiated by a leader, often (but not necessarily) with the intention to amuse the followers (Pundt and Venz, 2017). This definition suggests that the sender of humor—in this context: the leader—often has the intention to amuse his/her followers, when using humor. If humor unfolds its effects, the followers will be amused which has immediate positive consequences for affective states (e.g., Robert and da Motta Veiga, 2017). In the long run, humor in leadership is positively related to desirable outcomes such as performance, wellbeing, affective commitment, or innovative work behavior (see Kong et al., 2019, for a recent review).

The positive effects of humor may be explained by the relational process model by Cooper (2005, 2008). Following this model, leaders using humor trigger four relational processes. First, the immediately experienced positive affect after a humor event is generalized, which means that followers tend to assume that humorous events in interactions with their immediate leader might be a typical interaction pattern with their leader. Therefore, generalizing the positive experience of single humorous interactions, the followers might have a positive relationship expectation with their leader which will affect subsequent interactions positively. Based on affect generalization, a positive relationship between leader and follower is likely to develop.

The second relational process specified by Cooper (2005, 2008) is self-disclosure: Leaders using humor allow the followers to become acquainted with them on a deeper level of experience. Using humor in the context of leadership might be a leap of faith, in that the leaders disclose their true self and encounter their followers on a more personal and not merely professional level. This in turn might contribute to the development of a more positive relationship between leader and follower.

Third, humor unfolds its effects *via* the perception of similarity (Cooper, 2005, 2008). If humor succeeds, both the sender and the receiver of humor may share the experience of laughing or at least being amused about the same things. This in turn triggers the perception of similarity, which leads to higher levels of mutual liking and, subsequently, a more positive relationship between leader and follower.

Fourth, Cooper (2005, 2008) argues that humor might reduce the salience of hierarchical differences between leader and follower. By sharing humorous events, leaders allow for more personal encounters between themselves and their followers, which might lead to the perception that formally existing hierarchical differences between leader and follower are not that important in actual interactions between leader and follower. This impression might be an important step for the development of a positive relationship between leader and follower.

Empirical evidence has clearly demonstrated the relation between humor in leadership and the quality of the leader-follower relationship, which is often operationalized in terms of leader-member exchange (LMX; see Kong et al., 2019 for a recent meta-analysis). LMX is defined as the quality of the individual professional relationship between a leader and an individual follower (Graen and Uhl-Bien, 1995) and has shown to be an important mediator for the relationship between humor and several desirable outcomes (Kong et al., 2019). Although the evidence speaks for the relational process model, evidence so far is rather scarce on leaders’ use of humor with the *intention* to amuse their followers, but do *not succeed* in amusing followers. We try to shed some light on these situations by conceptualizing and initially investigating the concept of failed humor in leadership.

### Failed humor in leadership

In this paper, we define failed humor in leadership as the followers’ perception of a typical interaction pattern between leader and follower, which is typically characterized by frequent attempts of the leader to amuse the follower without causing the intended effect of amusement. We explicitly follow the terminology of Bell (2015), acknowledging that the term failed humor originally is a linguistic term bound to episodes of failed humor. Nevertheless, the term has been used in psychology as well (e.g., Williams and Emich, 2014), and therefore, we decided to stick with the term in order to demonstrate the continuity of our approach with previous research.

There might be several reasons for humor to fail its intended effects. Following the linguistic-pragmatic analysis by Hay (2001), there are four necessary steps toward humor to unfold its intended effect: First, the receiver must recognize a so-called humor-frame enabling him/her to distinguish between serious and humorous comments of the sender. Second, the receiver has to understand the humorous element. Third, the receiver has to appreciate the humor used by the sender, and fourth, the receiver has to agree to the message behind the humorous communication. On every step of this process, humor might fail (Dynel et al., 2016). This means, humor could fail, (a) because the receiver does not recognize the humor-frame, (b) because the receiver does not understand the humor, (c) because the receiver does actually understand but does not appreciate the humorous comment or, (d) the receiver does not agree to the message, even though the humor frame was clear, the humor was understandable, and the receiver even appreciated the joke. In either case, humor would fail to have the intended effect of amusing the receiver of humor.

Although Bell (2015) provides an even more differentiated view of reasons for humor to fail, the main point of her analyses seems to be that failed humor is a humorous communication which does not have the effect the sender intended. Therefore, and in line with Brock (2016), we define failed humor in leadership as discernibly intentional attempts of leaders to amuse their followers by utterances or behavior, without having the desired effect (e.g., mirth, cheerfulness, laughter) in a particular situation. In analyzing conversational patterns of humor, one typically finds so-called humor patterns, which means a humorous communication which is either followed by laughter or by another humorous communication (Lehmann-Willenbrock and Allen, 2014). From this point of view, failed humor would be an attempt of humorous communication by the leader which is not followed by laughter or other humorous communication. This means, failed humor is an isolated humorous attempt without being answered by the receiver and thus without leading to a typical humor pattern in the course of interaction.

It is important to note, that—different to the considerations of Dynel et al. (2016)—our definition is bound to humor recognized as such by the receiver, which, however, fails to have the intended effects. Therefore, the followers are aware of the humor frame, in which the leader intends to amuse the followers, however, without actually being amused. We emphasize this point with respect to our intention to explore the relevance of failed humor for the relationship between leader and follower. For having an effect on the relationship, failed humor has to be recognized as humor that has failed its effect. Although humor might also fail because the follower does not recognize the humor frame (Hay, 2001) or even because the follower did not understand the words spoken by the leader (Bell, 2015), failing in these cases would have no particular effect on the leader-follower relationship beyond the communication itself. We emphasize that for understanding the relational effects of failed humor in leadership, the most important condition is that the followers recognize the humorous communication as a humorous attempt, which, for whatever reasons, does not cause amusement on the side of the follower.

An important precondition for such a conceptualization of failed humor is that individuals have to be able to detect humorous intentions, even without having a complete humor pattern as used by Lehmann-Willenbrock and Allen (2014). Wyer and Collins (1992) argue that a humor frame is often recognized by non-verbal cues such as the facial expression or the jocular tone in the voice of the sender. Such cues are complemented by knowledge about the sender (e.g., knowing the “true” attitudes of the sender might help detect ironic comments; being familiar with the typical kind of jokes of a sender might help detect a humor frame even for unknown jokes). Thus, in a typical conversation between leaders and followers, followers should be very likely to be able to detect humorous attempts by the leader.

While one might be able to explore the immediate effects of a failed humor event in observational studies such as the one by Lehmann-Willenbrock and Allen (2014), we are more interested in the overarching effect of failed humor as an interaction pattern on the relationship between leader and follower. The relationship between leader and follower can be defined as a generalized interaction pattern typical for interactions between leader and follower (e.g., Asendorpf et al., 2017). As a consequence, we have to specify our definition toward a generalized interaction pattern between leader and follower that is characterized by perceptions of frequent humor attempts of the leader that typically fail to reach its intended effects.

In defining failed humor as the perception of a typical interaction pattern, it becomes clear that we refrain from defining failed humor as a humor style such as affiliative or aggressive humor (e.g., Martin et al., 2003). A humor style would be a typical and rather stable characteristic of the sender of humor. Failed humor, instead, is conceptualized as the perception of a typical interaction pattern—hence, it is rather a characteristic of the interaction between sender and receiver of humor. By using failed humor as such, we rather focus on the interplay between leader and follower with respect to humor attempts by the leader which are perceived as such without being perceived as funny.

### The relational effects of failed humor in leadership

If leader humor in general is strongly related to the leader-follower relationship, then it is important to show that failed humor is also relevant in explaining the leader-follower relationship. As of yet, research on the consequences of failed humor in the work setting is rather scarce. In one exception, Williams and Emich (2014) explored the consequences of failed humor for the sender of humor and were able to show that failed humor will lead to refraining from further humor attempts, a loss of (interpersonal) self-efficacy, and to withdraw from the relationship in general. Bell (2015) describes feelings of shame and humiliation that may result from failed humor attempts. It is plausible that a leader whose humor attempts typically fail, will perceive this situation as shameful and may refrain from other attempts to offer a positive professional relationship to the follower (Pundt and Herrmann, 2015). Therefore, we may assume that failed humor might lead to lower relational engagement on the side of the leader, which might be a reason for a negative association between failed humor in leadership and the quality of the leader-follower relationship.

On the side of the receiver, failed humor in leadership might have two different effects. On the one hand, the followers might appreciate the humor intention of the leader even though it does not have the effects humor typically has. If this was the case, failed humor might have positive effects on the relationship between leader and follower (Pundt and Venz, 2017). On the other hand, however, failed humor might cause feelings of dissonance between the discerned humor intention and the lack of humor effect. Dissonance is usually conceptualized as an uncomfortable state that leads to a motivation to reduce itself (Festinger, 1957). One solution for such dissonance reduction would be the conclusion that the relationship between leader and follower is of lower quality. Because leader and follower cannot laugh about similar things and are incompatible with respect to humor preferences, followers are not able to perceive similarity and thus to develop a more personal way of interacting with each other (Cooper, 2008). Failed humor might complicate the development of a positive professional relationship as followers, which typically starts with a relational offer sent by the leader, for example, *via* humor attempts (Pundt and Herrmann, 2015). When confronted with failed humor, followers might refrain from accepting this relational offer, even more so, if the pattern of failed humor is typical for the relationship between leader and follower. Therefore, we assume failed humor to be negatively related to leader-member exchange as a measure of the quality of the leader-follower relationship (Graen and Uhl-Bien, 1995).

***Hypothesis 1.*** Failed humor in leadership is negatively related to leader-member exchange.

### Incremental effects of failed humor in leadership

In establishing new constructs and new measures, it is important to demonstrate the incremental validity of the new construct beyond other, more established constructs in the field (Sechrest, 1963). Given the construct proliferation often lamented with respect to leadership research (Banks et al., 2016), demonstrating incremental validity is of particular importance. In the case of failed humor, affiliative, and aggressive humor in leadership might be regarded as well-established constructs. Affiliative and aggressive humor are two of the four humor styles postulated by Martin et al. (2003), which are particularly relevant for describing humor in leadership (e.g., Pundt and Herrmann, 2015; Robert et al., 2016). Affiliative humor is typically “used to enhance one’s relationships with others in a way that is relatively benign and self-accepting” (Martin et al., 2003, p. 52). Leaders with high levels of affiliative humor enjoy making their followers laugh and use their humor to enhance the relationship with their followers. In interactions with their followers, they easily and spontaneously think of witty comments and often laugh or joke when talking to their followers (Howland and Simpson, 2014; Pundt and Herrmann, 2015).

Aggressive humor in leadership refers to the leaders’ “hostile uses of humor, in which the self is enhanced by denigrating, disparaging, excessively teasing, or ridiculing others” (Martin et al., 2003, p. 52). It is a rather negative form of humor intended to mock other people. Leaders high in aggressive humor often use rough, dark, or sarcastic humor or socially inappropriate jokes when talking to their followers, often without even caring for the feelings such jokes might induce in their followers. Aggressive humor used by leaders is often intended to offend their followers or to express the leaders’ superiority to their followers (Howland and Simpson, 2014; Pundt and Herrmann, 2015).

Pundt and Herrmann (2015) were able to show that affiliative humor is related to a quality increase of the leader-follower relationship, whereas aggressive humor is related to a decrease of the relationship quality. With respect to the incremental validity of failed humor in leadership, we argue as follows: First, failed humor is more than the mere absence of successful affiliative humor, because the perception of the leader trying to use humor but failing to cause the intended effect carriers more information for the follower than the case of a leader not using affiliative humor in his/her leadership. We assume that failed humor may be a relationship warning signal for followers, and as this, it reaches beyond (a lack of) affiliative humor.

***Hypothesis 2.*** Failed humor in leadership incrementally explains variance in leader-member exchange beyond affiliative humor in leadership.

We also assume that failed humor is different to aggressive humor and should therefore, also explain incremental variance in LMX. While aggressive humor in leadership might be humiliating, annoying, or embarrassing for the follower, failed humor does not necessarily have these consequences. Rather, it leads to secondhand embarrassment on the side of the follower who observes an embarrassingly failing humor attempt on the side of the leader. Moreover, we assume that aggressive humor in leadership will cause a lack of motivation of the follower to let in the relational offers by the leader, while failed humor will rather cause a lack of possibility to let it the leader’s relational offers.

***Hypothesis 3.*** Failed humor in leadership incrementally explains variance in leader-member exchange beyond aggressive humor in leadership.

## Materials and methods

### Sample and procedure

For this study, we combined two independent data sets. Both samples are convenient samples. Sample 1 was collected by the third author of this study, while Sample 2 was collected by the second author of this paper. After combining the data sets, the overall sample consists of 385 employees, 235 women (61.0%) and 150 men (39.0%). We describe each data collection separately because of slight differences between the respective procedures.

For sample 1, we conducted a web-based survey study using SoSci Survey (Leiner, 2019). Participants were recruited *via* social media and from the pool of acquaintances of the third author of this study, targeting people working in an organization and having an immediate leader. Overall, 125 people finished the questionnaire. We excluded two respondents from the survey, because they self-reported to just have clicked through the survey without sufficient attention (check item), and three more respondents because they did not fulfill the criterion of having an immediate leader because they were self-employed.

The remaining sample consists of 120 employees from various organizations, 89 of which (74.2%) were female and 31 of which (25.8%) were male. Respondents were in the age ranging from 18 to 75 (*M* = 27.47, *SD* = 10.99), and they had a high level of education with 66.7% having a high school degree and 22.5% a university degree. The respondents were employed in various sectors of industries with larger shares of respondents working in the human health and social work sector (22.5%), in the accommodation and food service sector (18.3%), in the service sector (combination of professional, scientific, and technological services, administrative/support services, and other services, 19.8%), in the arts, entertainment, and recreation sector (8.5%), in the trading sector (7%), and in public administration (7%). The shares of respondents working in other sectors were smaller than 6% each.

Sample 2 was also collected *via* a web-based survey, this time using Unipark. Similar to sample 1, participants were recruited *via* social media and from the pool of acquaintances of the second author of this study. As in sample 1, we targeted people working in an organization and having an immediate leader. Overall, 284 people took part in the survey. We had to exclude 19 respondents from the survey, because they did not fulfill the criterion of having an immediate leader because they were self-employed or owners of a business. We also excluded four respondents due to implausible long run times of the survey, which might indicate interruptions or distractions during answering the survey.

The remaining sample consists of 265 employees from various organizations, 146 of whom (55.1%) were female, and 119 (44.9%) were male. Respondents were in the age range from 18 to 65 years (*M* = 32.69 years, *SD* = 13.02 years). The educational level of the respondents was rather high, with 33.6% having a university degree, 36.2% having a high school degree, and 22.3% having vocational training. Participants had a contractual working time of 29 h per week on average (*SD* = 11.81).

More than half of the participants (50.6%) worked in organizations that employed up to 249 employees, 31.3% worked in organizations with more than 1,000 employees and the remaining 18.1% worked in organizations with between 250 and 1,000 employees. Larger shares of respondents worked in the human health and social work sector (22.4%), in the manufacturing sector (15.3%), in the service sector (professional/scientific/technological services, administrative/support services, and other services combined, 13.6%), in the trading sector (9.6%), in the information and communication sector (8.9%), and in the accommodation and food services sector (8.5%). The shares of respondents working in other sectors were smaller than 6% each.

A share of 31.3% of the respondents reported their immediate leader to be female, 68% worked for a male leader, and 1 person (i.e., 0.4%) categorized his/her immediate leader as diverse. Nearly 50% of the respondents reported to have direct contact with their leaders on a daily basis, with 41.5% of them having direct contact more than once a day. Additional 31.3% reported having direct contact with their leaders 2–3 times per week, and 19% had only infrequent direct contact with their immediate leader (1–2 times per month or less). Because the contact frequency may be relevant for the opportunity to observe (failed) humor in leadership, we decided to include contact frequency as a control variable.

### Measures

*Failed humor* was measured with a newly developed 12 item scale. Items formulation was based on the definition given above. In order to set the frame of situations in which the leader attempts to use humor, all of the items were starting with the sequence “If my immediate leader tries to be humorous….” The single items were then ended with phrases such as “…he/she is not really funny” or “…often nobody can laugh about it” in order to represent the failing of the leaders’ humor attempts. For the full scale, please see in Table 1. Respondents answered these items on a 5-point rating-scale from 1 (*do not agree at all*) to 5 (*do completely agree*).

**TABLE 1:** Failed humor scale.

	Original German item wording	English item wording
	**Wenn meine Führungskraft versucht lustig zu sein…**	**When my leader is trying to be funny,…**
01.	**… ist sie dabei nicht wirklich witzig.**	**… they are not really funny.**
02.	**…** geht das oft nach hinten los.	**…** it often backfires.
03.	**… kann oft niemand darüber lachen.**	**… often no one can laugh at it.**
04.	**… verunsichert mich das eher, als mich zum Lachen zu bringen.**	**…it unsettles me rather than makes me laugh.**
05.	**…** geht das oft daneben.	…it often misses the mark.
06.	**…** entsteht oft ein peinliches Schweigen.	**…** an awkward silence often ensues.
07.	**… ist das meistens nicht amüsant.**	**…it’s usually not amusing.**
08.	**…** geht der Witz manchmal unter.	…sometimes the joke gets lost.
09.	**…** führt das selten zum gewünschten Effekt.	…it rarely leads to the desired effect.
10.	**…** lache ich manchmal nur aus Höflichkeit.	…I sometimes laugh just to be polite.
11.	**… erzeugt das nicht mehr als ein müdes Lächeln**.	…**it doesn’t generate more than a weary smile**.
12.	**… erkennt man den Scherz, findet ihn aber meistens nicht lustig.**	**…you can tell the joke, but you usually don’t find it funny.**

Items of the short six-item scale in boldface.

The item analysis showed mean values for all items between 1.96 and 2.75 and standard deviations between 0.97 and 1.24. This means that, although the mean level of responses is not particularly high, we are able to observe substantial differences between people in responding to these items. The item-total correlations were rather high for all items and ranged between 0.60 and 0.81.

A subsequently conducted exploratory factor analysis (principal axis factor analysis) showed one factor with an eigenvalue above 1 (Guttman, 1954; Kaiser and Dickman, 1959). The extracted factor had an eigenvalue of 7.23 thereby explaining 60.28% of the variance. The scree plot (Cattell, 1966) showed a clear leveling off in the eigenvalues with the second factor having an eigenvalue of 0.838 (to demonstrate the leveling off: the third and fourth factor have eigenvalues of 0.725 and 0.566). The factor loadings of the items ranged between 0.63 and 0.83. Internal consistency of the original 12-item scale was α = 0.94.

In addition, we calculated a confirmatory factor analysis in order to examine whether the newly created scale is unidimensional in its factorial structure. The resulting fit indices show an acceptable fit with respect to the Standardized Root Mean Square Residual (SRMR) = 0.041, however, with respect to χ^2^ = 270.778, df = 54, *p* < 0.01, χ^2^/df = 5.014, Comparative Fit Index (CFI) = 0.930, Tucker Lewis Index (TLI) = 0.914, or Root Mean Square Error of Approximation (RMSEA) = 0.102, the fit is barely acceptable (Hu and Bentler, 1999). Therefore, we went one step further and tried to establish a shortened scale with a better fit.

Soto and John (2019) investigated the optimal scale length of short scales. They argue that while classical as well as probabilistic test theory would imply longer scales to be more valid, the relation between scale length and validity is not necessarily linear. Instead, they found a diminishing marginal utility of increases in item numbers of a scale: “As the length of a scale increases, each additional item will provide a proportionally smaller boost to measurement precision and, thus, to validity” (Soto and John, 2019, p. 445). In their study, they find scales with 6–9 items to nearly completely reach the validity level of 12-item scales. In other words: A short scale of six items might be comparably valid as a 12-item scale, and would, however, have the advantage of less administrative effort in terms of response time and fatigue of respondents.

We used an automated item selection algorithm to create a unidimensional six-item instrument. Since algorithmic approaches are not yet common practice in organizational and social sciences, we give a brief overview. Traditionally, psychological instruments are abbreviated by selecting items that simultaneously maximize item-total correlations and maintain high internal consistency (Yarkoni, 2010). An optimal approach, however, requires researchers to select items not only based on their unique qualities, as classical approaches do, but to improve the psychometric properties of a set of items given a predefined set of constraints (Stanton, 2000; Schultze, 2017).

Developing or adapting an instrument, i.e., selecting items to create a psychometrically sound instrument, can be defined as a combinatorial problem (Kerber et al., 2019). Combinatorial problems, such as the *knapsack problem* (Schroeders et al., 2016, p. 4) refer to the process of finding a discrete and finite solution given a set of constraints (Hoos and Stützle, 2004). In the context of constructing psychological assessment instruments, the problem can be understood as selecting a set of items from an original item pool that fulfills certain predefined criteria (e.g., selecting six items to create a short instrument with good model fit).

Besides the classical approaches to scale development and adaptation (e.g., confirmatory factor analyses), contemporary approaches use automated item selection algorithms. These so-called meta-heuristics are particularly useful because the psychometric criteria can only be computed in combination with other items, with the aim to improve the quality of the scale as a whole (Olaru and Danner, 2021). Recent findings in scale development or adaptation suggest that algorithmic approaches perform at least as well as traditional approaches (Sandy et al., 2014) or even outperform them (Schroeders et al., 2016; Olaru and Danner, 2021).

#### Item selection procedure

We used the “*bruteforce”* function of the R package “*stuart”* version 0.9.1 (Schultze, 2020), which computes all possible item combinations to obtain the single best solution. Since choosing six items from a 12-item scale results in 924 possible solutions, this can be achieved in a short amount of time and at low computational cost. The original data set was randomly split into a training (*n*_1_ = 193) and a test data set (*n*_2_ = 192). The solutions were evaluated against an objective function consisting of a combination of the model fit criteria RMSEA, SRMR and the CFI as well as a composite reliability computed as McDonald’s omega (ω). In the next step we cross-validated our findings with the test data set using the “*crossvalidate*” function of the R package “*stuart*” (Schultze, 2020).

#### Evaluation of model fit, measurement invariance and external validity

Model fit is evaluated using standard recommendations proposed by Hu and Bentler (1999). These comprise of χ^2^ significance testing as well as a combination of several fit indices, i.e., RMSEA < 0.05, SRMR < 0.07, CFI > 0.95. The CFA is run with the R package “*lavaan*” (Rosseel, 2012). Furthermore, the selected scale is cross-validated, in order to examine whether the solution holds in a test sample with regard to the four standard measurement invariance assumptions based on Meredith (1993).

The selected solution exhibits good model fit with Satorra-Bentler- χ^2^(9, *N* = 193) = 6.30, *p* = 0.71, CFI = 1.00, SRMR = 0.01, RMSEA = 0.00, 90%-CI_*RMSEA*_ (0.00; 0.04). All items loaded statistically significantly on the factor *failed humor.* Standardized loadings ranged from 0.67 to 0.84. All factor loadings including standard errors can be found in Table 2. McDonalds ω of the failed humor scale was 0.89. Cross-validation with the second half of the data indicated that the assumption of strict measurement invariance holds across the two subsamples: χ^2^(35, *N* = 192) = 49.14, *p* = 0.06, SRMR = 0.04, RMSEA = 0.04, Δχ^2^ = 8.02, Δ *df* = 6, *p* = 0.24.

**TABLE 2:** Standard errors and standardized factor loadings of the failed humor scale.

Item	SE	Std.all
1 … **they are not really funny**		0.704
3 … **often no one can laugh at it**.	0.073	0.767
4 … **it unsettles me rather than makes me laugh**.	0.082	0.667
7 … **it’s usually not amusing**.	0.088	0.835
11 … **it doesn’t generate more than a weary smile**	0.084	0.797
12 … **you can tell the joke, but usually don’t find it funny**.	0.085	0.841

All items are started with “If my immediate leader tries to be funny….”

*Affiliative humor and aggressive humor* were measured with the respective subscales of the Humor Styles Questionnaire (HSQ) by Martin et al. (2003). In line with Pundt and Herrmann (2015), the items were formulated in a way that the respondents rated their immediate leader. A sample item of the eight-item scale measuring affiliative humor was “My leader enjoys making people laugh.” a sample item for the aggressive humor scale (also eight items) was “If my leader does not like someone, he/she often uses humor or teasing to put them down.” Respondents answered the item on a five-point rating-scale from 1 (*never or very seldom*) to 5 (*very often*). Cronbach’s Alpha for the affiliative humor scale was 0.89 and 0.71 for the aggressive humor scale. The rather low internal consistency of the aggressive humor scale was mainly due to rather low item-scale intercorrelations of two items (item 4 and item 6)—if deleting these items, the internal consistency was growing to 0.75. However, in order to sustain comparability with previous studies, we decided to analyze our data with the full aggressive humor scale thereby accepting the rather low level of internal consistency, even more so, as rather low internal consistencies of the aggressive humor scale have been found in several studies before (e.g., Martin et al., 2003; Wisse and Rietzschel, 2014; Pundt and Herrmann, 2015; Kim et al., 2016; Robert et al., 2016; Scheel et al., 2016). *Leader-member exchange* was measured with the seven-item scale by Graen and Uhl-Bien (1995; German version by Schyns, 2002). The scale consists of seven questions with varying answering options. A sample item is “How well does your leader recognize your potential?” Respondents answered the questions on 5-point rating-scales—for the sample item from 1 = “*not at all*” to 5 = “*fully*.” Internal consistency (Cronbach’s Alpha) was 0.90.

#### Discriminant validity

In order to examine the discriminant validity of the failed-humor scale, we conducted further CFAs and tested a four-factor model with failed humor, affiliative humor, aggressive humor, and leader-member exchange as latent variables. We allowed for intercorrelations between the latent variables, however, we did not allow for correlations between error terms. The fit indices show an overall acceptable model fit, with χ^2^ = 1,024.850, df = 371, χ^2^/df = 2.762, RMSEA = 0.068, SRMR = 0.070, except for CFI = 0.885, TLI = 0.875. Although the fit is not optimal, we assume that this is due to two items of the aggressive humor scale which had rather low item-total correlations in our sample. Actually, excluding these items would have led to a better fit with χ^2^ = 726.414, df = 318, χ^2^/df = 2.284, RMSEA = 0.058, CFI = 0.925, TLI = 0.917, SRMR = 0.061. However, as it is not our aim to optimize established scales in the first place, we decided to not change any scale besides the new scale for failed humor in order to sustain comparability with previous research.

We then compared the original four-factor model with a three-factor model combining failed humor and aggressive humor in leadership to one factor, resulting in a poorer fit than the four-factor model, with χ^2^ = 1,437.551, df = 374, χ^2^/df = 3.843, Δχ^2^(3) = 412.701, *p* < 0.01, RMSEA = 0.081, CFI = 0.813, TLI = 0.798, SRMR = 0.093. We also compared the four-factor model with a two-factor model combining the three humor scales affiliative, aggressive, and failed humor to one factor, and found an even poorer fit than for the three factor model with χ^2^ = 2,271.716, df = 376, χ^2^/df = 6.041, Δχ^2^(2) = 1,246.866, *p* < 0.01, RMSEA = 0.114, CFI = 0.668, TLI = 0.641, SRMR = 0.110. Overall, these analyses show that the scale we created for measuring failed humor in leadership shows sufficient discriminant validity with respect to affiliative and aggressive humor in leadership.

## Results

Table 3 shows the descriptive statistics and intercorrelations for all scales used in this study. In line with Hypothesis 1, we found a negative correlation between failed humor in leadership and leader-member exchange (*r* = −0.61, *p* < 0.001). Moreover, we found failed humor to be negatively correlated with affiliative humor (*r* = −0.53, *p* < 0.001) and to be positively correlated with aggressive humor (*r* = 0.29, *p* < 0.001).

**TABLE 3:** Descriptive statistics and intercorrelations between study variables.

	*M*	*SD*	α	1	2	3
1. Failed humor	2.36	0.89	0.90	−		
2. Affiliative humor	3.41	0.84	0.89	−0.53**	−	
3. Aggressive humor	2.66	0.73	0.71	0.29**	0.03	−
4. Leader-member exchange	3.56	0.79	0.90	−0.61**	0.49**	−0.35**

N = 385, **p < 0.01.

In order to test our Hypotheses 2 and 3, we conducted a series of hierarchical regression analyses, the results of which are shown in Table 4. In Model 1, we regressed leader-member exchange on affiliative humor and failed humor. We found affiliative humor to be positively related to leader-member exchange (β = 0.24, *p* < 0.01). Failed humor in leadership incrementally predicted leader-member exchange beyond affiliative humor (β = −0.48, *p* < 0.01, Δ*R*^2^ = 0.17). In Model 2, we regressed leader-member exchange on aggressive humor and failed humor. While aggressive humor was negatively related to leader-member exchange (β = −0.20, *p* < 0.01), we found failed humor to incrementally predict leader-member exchange beyond aggressive humor (β = −0.55, *p* < 0.01, Δ*R*^2^ = 0.28). In Model 3, we regressed leader-member exchange on affiliative and aggressive humor as well as on failed humor. We found affiliative humor to be positively related (β = 0.31, *p* < 0.01) and aggressive humor to be negatively related to leader-member exchange (β = −0.26, *p* < 0.01), and we found failed humor to incrementally predict leader-member exchange beyond both affiliative and aggressive humor (β = −0.37, *p* < 0.01, Δ*R*^2^ = 0.09).

**TABLE 4:** Multiple regression analyses of leader-member exchange on humor in leadership.

	Model 1		Model 2		Model 3	
	Step 1	Step 2	Step 1	Step 2	Step 1	Step 2
Affiliative humor	0.49**	0.24**			0.50**	0.31**
Aggressive humor			−0.35**	−0.20**	−0.37**	−0.26**
Failed humor		−0.48**		−0.55**		−0.37**
*R* ^2^	0.24**	0.41**	0.13**	0.40**	0.38**	0.47**
Δ*R*^2^		0.17**		0.27**		0.09**
*F*	123.89	132.16	54.47	128.26	116.13	110.74

N = 385, standardized β-coefficients, **p < 0.01.

In addition to our hypotheses testing, we conducted a relative weight analysis as suggested by Tonidandel and LeBreton (2015, see also Tonidandel and LeBreton, 2011). We used the web-application RWA Web Shiny Apps (2011), which is a new version of the application suggested by Tonidandel and LeBreton (2015). The results showed a raw relative weight of 0.22, CI95% (0.16, 0.28) for failed humor as compared relative weights of 0.16, CI95% (0.11, 0.21) for affiliative humor and 0.09, CI95% (0.05,0.14) for aggressive humor. This means that all failed humor, affiliative humor, and aggressive humor are all of importance in predicting leader-member exchange. As the confidence intervals of the relative weights of failed humor and of aggressive humor do not overlap, the difference between the relative weights of failed and aggressive humor can be interpreted as statistically significant. Expressed in terms of rescaled relative weights, failed humor explains 46.78% of all variance in leader-member exchange explained by the predictors (*R*^2^ = 0.47), while affiliative humor and aggressive humor have a rescaled relative weight of 33.50 and 19.71%, respectively. Therefore, our findings show that failed humor seems to have the strongest weight in predicting leader-member exchange as compared to affiliative and aggressive humor.

## General discussion

In this study, we investigated the relationship between failed humor in leadership and leader-member exchange. Building on previous attempts to capture the essence of failed humor (Williams and Emich, 2014; Bell, 2015; Brock, 2016), we conceptualized failed humor in leadership as the followers’ perception of a generalized interaction pattern between leader and follower that is characterized by humor attempts of the leader which typically fail to reach its intended effects. We developed a new scale for measuring failed humor in leadership with adequate psychometric properties. Furthermore, we found initial evidence for a negative relation between failed humor in leadership and leader-member exchange, hence supporting the idea that relational mechanisms cannot unfold when humorous attempts of the leaders typically fail to reach their intended effects. Moreover, we found evidence for failed humor to have incremental validity beyond established humor concepts such as affiliative and aggressive humor, which underlines the necessity of the newly developed construct.

### Theoretical implications

Our findings have several theoretical implications. First, our findings underline the necessity of investigating the failing of humor in leadership more closely. As we found failed humor to uniquely predict variance in leader-member exchange even beyond established humor constructs, we are able to show that the failing of a leader’s humor is different from the leader just having low values in humor behavior. It seems as if failing attempts of humor that are perceived as typical for the relationship between leader and follower are even more detrimental for the leader-follower relationship than just the lack of affiliative humor or even the presence of aggressive humor. Our finding therefore underlines the notion raised by Bitterly (2022, see also Bitterly et al., 2017) that using humor might be risky and adds a new risk to the discussion: humor might fail, at least in the eye of the receiver, and this might be a risk for the leader as the sender of humor.

In line with establishing failed humor as a unique construct, our study implies that there might be a gap between humor attempts or humor intentions, on the one hand, and the effect that is actually reached with the humor attempt on the other hand. Research on humor in leadership so far is based on the implicit assumption that humor attempts by the leaders are (more or less) successful in terms of amusing followers. However, our findings support common wisdom by which humor attempts of a leader are by no means predetermined to amuse followers. Therefore, we can conclude that investigating failed humor in addition to successful humor in leadership might enhance our knowledge of the role of humor in leadership and complete the theoretical picture we have of humor in leadership.

Our findings also show that the relational process model of humor in leadership (Cooper, 2008) needs to be expanded with respect to the possibility that humor attempts by the leader fail to have the intended effects. Though the model explains what will not happen in case of failing humor (e.g., no affect reinforcement, no self-disclosure, no perception of similarity, no reduction of hierarchy salience), the not-setting-in-motion of these relational processes might not be sufficient in explaining the effects of failed humor, because these processes would not be set in motion by a simple lack of affiliative humor. However, as our findings show, failed humor incrementally predicts leader-member exchange even beyond (a lack of) affiliative humor. This raises the question of which mechanism might be relevant in explaining the detrimental effects of failed humor on leader-member exchange.

### Limitations and implications for future research

One obvious limitation of our study is that we collected data in a cross-sectional survey. The use of non-probability samples in this study raises further concerns about generalizability. Hence, our results should be interpreted as rather initial evidence of the possible effects of failed humor. Of course, it is not possible to draw causal conclusions from our data. However, given our aim of empirically exploring the concept of failed humor in leadership and developing and validating a scale for measuring failed humor in leadership, a cross-sectional design might still be adequate. However, although cross-sectional design is legitimate in a first validation study, future studies are needed in order to actually demonstrate the effect of failed humor on leader-member exchange in a longitudinal study, as, for example, has been done by Pundt and Herrmann (2015).

In our study, we focused on a rather general assessment of failed humor in leadership. Although this is intended, because we think that the effect would only unfold if the failing of humor is perceived to be a typical interaction pattern (Asendorpf et al., 2017), further studies should look more closely at particular events of failed humor in daily leader-follower interactions and compare the results with more general measures. Such studies might be conducted as event sampling studies asking for failed humor directly after each interaction between leader and follower. Alternatively, observational studies could be used (e.g., Lehmann-Willenbrock and Allen, 2014). Such studies, however, would have to deal with the difficulty of observing humor attempts by the leader without seeing the followers’ amusement or emerging sequences of humor as the result of such attempts (which would be *failed* humor). Research would thus need a deeper understanding of how to observe humor attempts even without them having an effect on the follower’s amusement. One possible way might be an observational study including the retrospective comments by the participants of a meeting.

In a related way, it might also be interesting to see whether independent observers might come to similar conclusions with respect to failed humor in leadership. Conceptually, however, it would not threaten the validity of our concept of failed humor, if independent observers would not agree with respect to failed humor, because we view failed humor in leadership as the perception of a typical interaction pattern between leader and follower. Such a concept is conceptually similar to abusive supervision (e.g., Tepper, 2000) which is also defined in terms of follower perceptions of hostile behaviors of the leader. It is per definition that independent raters do not have to come to similar judgments when rating failed humor in leadership. Nevertheless, as in research on abusive supervision (cf. Martinko et al., 2013), it would be important to disentangle the sources of variance with respect to failed humor in leadership.

In order to further establish construct validity of our measure, it might also be interesting to see whether failed humor in leadership is related to other humor constructs such as self-defeating humor or self-enhancing humor (Martin et al., 2003) or the humor styles (e.g., competent vs. inept humorous style as suggested by Craik et al., 1996). However, it is important to not only build nomological networks in the context of humor concepts but also in the context of leadership. Investigations of the construct validity of failed humor in leadership should therefore also take more general leadership concepts such as transformational leadership (e.g., Bass, 1985) or abusive supervision (Tepper, 2000) into account.

Future research should also explore the impact of one-time experiences of failed humor, for example with respect to its impact on the perception of following humor events. Based on the idea of the Wheel Model of humor (Robert and Wilbanks, 2012), one might speculate that failed humor might lead followers to look more critically at future humor attempts of their leader, which might lead into a downward spiral with respect to the leader-follower relationship. It would be of high theoretical interest to see whether there are some relational trajectories that are driven by humor in general and failed humor in particular.

Given the need to distinguish between humor intentions and humor effect, one further step for future research might be investigating a form of leaders’ humor that has positive effects on follower amusement despite being unintended. Such forms of humor might be called involuntary humor (e.g., Wyer and Collins, 1992; Martin, 2007; Brock, 2016). The effect of involuntary humor in the leadership context is yet completely unclear and might deserve some attention. Classical experiments on the pratfall effect (Aronson et al., 1966) might guide such research.

### Practical implications

Given the exploratory character of this study, it is not very straight-forward to derive practical implications. Clearly, more research is needed to support our claim that failed humor is an important and relevant construct that leadership researchers and leadership practitioners should be concerned with. Assuming that future research might support this claim, some preliminary implications for leadership practice might still be derived. The first and maybe most obvious implication would be that leadership should be aware to avoid failed humor because of its detrimental effects on the leader-follower relationship. For avoiding failed humor, it would be safest to completely forgo using any humor in leadership. However, if leaders avoid using any humor in leadership, they would also forgo the many positive effects of successful humor (e.g., Kong et al., 2019). A better solution would therefore be to still use humor in leadership while being aware that humor might fail.

In our study, we conceptualized failed humor as a generalized interaction pattern as perceived by the followers. We showed that such interaction patterns might have negative effects on the leader-follower relationship. Nevertheless, we would assume that not every single event of failed humor would have detrimental impact on the leader-follower relationship (see also Williams and Emich, 2014). Therefore, it might be more interesting to develop strategies of competently dealing with single failing attempts of humor in order to avoid the perception of a generalized interaction pattern. Williams and Emich (2014) show that the way how leaders typically deal with failed humor is not very constructive and might lead them into a negative spiral of losses of relationship quality. They point at the importance of persistence in the willingness of leaders to interpersonally regulate the followers’ emotions, for example, by using humor in leadership. Williams and Emich (2014) argue that the leaders’ emotional perspective taking, and self-efficacy predict the persistence of using humor even after failed humor attempts and help leaders overcome the feelings of guilt and shame after failed-humor events.

Another strategy of dealing with failed-humor events might be the use of self-defeating humor. Self-defeating humor is defined as an “excessively self-disparaging humor, or attempts to ingratiate oneself or gain the approval of others by doing or saying funny things at one’s own expense” (Martin et al., 2003, p. 52). While self-defeating humor was originally “seen as potentially detrimental to wellbeing when used excessively” (Martin et al., 2003), later research has shown that the use of self-defeating humor is not that detrimental when not used very frequently (e.g., Caird and Martin, 2014). One might speculate here, that using self-defeating humor in order to cope with a single failed-humor event might have short-term advantages—it might be helpful to use self-defeating remarks when noticing an attempt of failing humor. Obviously, there might be some parallels between self-defeating humor and failed humor. In more general terms, it might be important to include dealing with failed humor attempts in general communication training or in specialized humor training.

In the long run, the aim of such training should be to develop a sense of how followers perceive the humor attempts by leaders and to reflect the effects that these attempts might have on the follower. The most important step is becoming aware of situations in which the leader tries to be funny without having the effect on followers and to learn how to adapt his/her humor attempts to the respective follower. In a more global way, it is a question of perspective taking (Gregory et al., 2011) and being self-aware (Tekleab et al., 2008) in order to avoid failed humor and, overall, to become a better leader (Sosik and Jung, 2010).

## Data availability statement

The raw data supporting the conclusions of this article will be made available by the authors, without undue reservation.

## Ethics statement

Ethical review and approval was not required for the study on human participants in accordance with the local legislation and institutional requirements. The patients/participants provided their written informed consent to participate in this study.

## Author contributions

AP contributed to conception and design of the study and wrote the first draft of the manuscript. AP, JK, and KH organized the database. AP, MA, and TL performed the statistical analysis. All authors contributed to manuscript revision, read, and approved the submitted version.

## Conflict of interest

The authors declare that the research was conducted in the absence of any commercial or financial relationships that could be construed as a potential conflict of interest.

## Publisher’s note

All claims expressed in this article are solely those of the authors and do not necessarily represent those of their affiliated organizations, or those of the publisher, the editors and the reviewers. Any product that may be evaluated in this article, or claim that may be made by its manufacturer, is not guaranteed or endorsed by the publisher.
